# Analysis of the characterization of NaOH-treated natural cellulose fibre extracted from banyan aerial roots

**DOI:** 10.1038/s41598-023-39229-9

**Published:** 2023-08-03

**Authors:** Raja Thandavamoorthy, Yuvarajan Devarajan, Subash Thanappan

**Affiliations:** 1 Material Science Lab, Department of Prosthodontics, Saveetha Dental College and Hospitals, SIMATS, Chennai, Tamilnadu India; 2 Department of Mechanical Engineering, Saveetha School of Engineering, SIMATS, Chennai, Tamilnadu India; 3 Department of Civil Engineering, Ambo University, Ambo, Ethiopia

**Keywords:** Environmental sciences, Mechanical engineering

## Abstract

Natural fibre is renewable and extensively utilized for structural and medicinal applications. The current research concentrates on surface modification for fibre enhancement using an alkaline treatment technique to extract raw fibre from banyan (*Ficus benghalensis*) aerial root bark. Using a 10% NaOH solution, attempts have been made to improve the crystalline, surface, thermal, physical, and chemical properties of banyan aerial root fibre (BAF). Five samples of BAF were produced by soaking the unprocessed fibre in an alkaline solution for variable amounts of time. On the surface of the treated BAF, a higher concentration of cellulose could be seen. The X-Ray Diffraction test revealed that the crystallinity index improved by 52%, with a crystalline dimension of 51.2 nm. It was observed that the crystalline content is increased in treated Banyan aerial root fiber due to this alkali treatment. The significance of natural fibre characterization is also briefly discussed, and this summary will serve as a resource for future studies on natural fibre composites by other researchers.

## Introduction

Reinforcement of thermoplastic and thermoset polymers constitutes the bulk of chemical treatments for natural fibres. An increase in surface roughness and fibre strength results from breaking hydrogen bonds in the fibres' network structure^1^. The fibres are bolstered during this process. Adding NaOH to natural fibres converts the hydroxyl group to an alkoxide ion. More and more people are turning to natural fibre-reinforced polymer composite because of its wide range of features^2^. Natural fibres are gaining popularity due to their many desirable characteristics, including their low cost, biodegradability, recyclability, non-abrasiveness, combustibility, lightweight, and lack of toxicity. The processing of raw materials and the construction of composite structures, while still complex, require further fundamental understanding^3^.

There are many places in the globe to harvest natural fibres from various animals, plants, and even minerals. Extraction procedures and various processing techniques affect the quality of natural fibres^4^. Natural fibre-reinforced composites, intended as a drop-in replacement for glass fibre composites, have been the subject of experimental development. The FTIR, XPS, and ESEM were used to characterize natural fibres' untreated and treated surfaces^5^. The removal of hemicellulose and lignin from natural fibre surfaces was evidenced by shifts in the peaks at 1730, 1625, and 1239 cm^−1^ in the FTIR spectrum after the alkali treatment. ESEM analysis of treated hemp and kenaf revealed the presence of silane. The surface qualities of these natural fibres might be improved by applying an appropriate surface treatment and chemical treatment^6^. Fibres treated in this way absorb less water, become stickier and boost the polymer composites' overall performance^7^. Alkaline therapy (NaOH) is the most used chemical treatment since it is both practical and inexpensive. Classification, composition, structure, characteristics, extraction techniques, chemical and surface treatments, and more are all proposed for natural fibres in this review paper. We also summarise the results of past studies on the fibre treatment, characteristics, and applications of natural/natural hybrid polymer composites and natural/synthetic hybrid polymer composites^8^. This research aimed to evaluate the viability of using Etlingera elatior stalks as a source of natural fibres. The stalk was submerged in water for 32 days to get the fibres, known as "water-retting." With the results of the tests in hand, the fibre's potential for spinning into yarn was assessed^9^. The Etlingera elatior fibre’s FT-IR spectra show cellulose, hemicellulose, and lignin. It was calculated that the crystallinity index was 43.93%.

Lots of little fibres make up a bundle of *Etlingera elatior*. Surface irregularities and roughness were seen in the fibre^10^. The fibres' cross-sections come in a wide range of sizes and shapes, from perfectly round to semi-elongated to oval. A 32.76 g/Tex tensile strength and an 11.8 per cent elongation were measured for the fibres. The fibres achieved a fineness of 6.67 Tex. The average height was 121.30 inches in length. *Etlingera elatior* fibres are hygroscopic, retaining 11.79% of their original moisture upon drying. The average friction coefficient of the fibres was 0.2. In terms of fibre qualities, *Etlingera elatior* fibres are suitable for spinning. The *Etlingera elatior* plant's extracted natural fibres may find a purpose in the textile industry^11^. The effect of various surface treatments on the thermo-mechanical and water-absorbing capabilities of coconut tree peduncle fibre-reinforced polymer composites. The potassium permanganate-treated CTPF composite is suitable for lightweight mobility and structural applications due to its maximum tensile strength of 128 MPa, flexural strength of 119 MPa, impact strength of 9.9 J/cm^2^, hardness value of 99 HRRW, and thermal stability up to 193 °C^12^.

New materials have been created in recent decades to enhance human well-being. Creating plate materials to fix broken bones is crucial since bone fractures are common. Material for these plates should be light, safe for human tissue, and rigid enough to do its job. Renewable in nature, natural fibres also have a higher perceived value in the marketplace^13^. For example, sisal, banana, and roselle are often used as reinforcement in India, all of which come from the Asian markets^14^. This study details the moulding process used to create plates from powdered natural fibres such as sisal (Agave sisalana), banana (Musa sapientum), and roselle (Hibiscus sabdariffa) using bio-epoxy resin Grade 3554A and Hardener 3554B. The current study focuses on predicting the flexural rigidity of the NFRP composite and comparing that prediction to the results obtained from the ANSYS solution^15^. A consensus is reached that they agree on most points. A scanning electron microscope is used to examine the material's microstructure. The study's primary goal was to find ways to put renewable resources to use in the creation of biocomposite materials from biopolymers and natural fibres. Externally covered with calcium phosphate and hydroxyapatite (hybrid) composite, this plate material can be employed for internal fixation and external fixing of fractured bones in the future^16^. Identify the results of the sorption and dielectric properties of woven jute after being treated with an alkali solution. After being subjected to alkali treatment, fibres were shown to have improved moisture sorption capabilities and a higher ability to store water. Seed and bark fibres were studied for their potential as a new material for fibre-reinforced composites. The research showed that compared to seed fibres, bark fibres included more cellulose and less lignin and extracted fibres^17^.

Inference from the above studies concludes that the surface modification shall improve the banyan fibre's physical and chemical characteristics. The unique fibre was recovered from the banyan aerial root, implying that the tree was not torn down to create this new fibre. As a result, this banyan aerial root fibre can lessen negative environmental effects while improving natural fibre reinforcement in fabricating polymer composite products. Hence, this work attempts to enhance the crystalline, surface, thermal, physical, and chemical characteristics of banyan aerial root fibre (BAF) using a 10-weight per cent NaOH solution and further analyzed by SEM, EDX, XRD, FTIR, TGA, tensile strength, and particle size.

## Materials and experimental procedure

**Figure 1:**
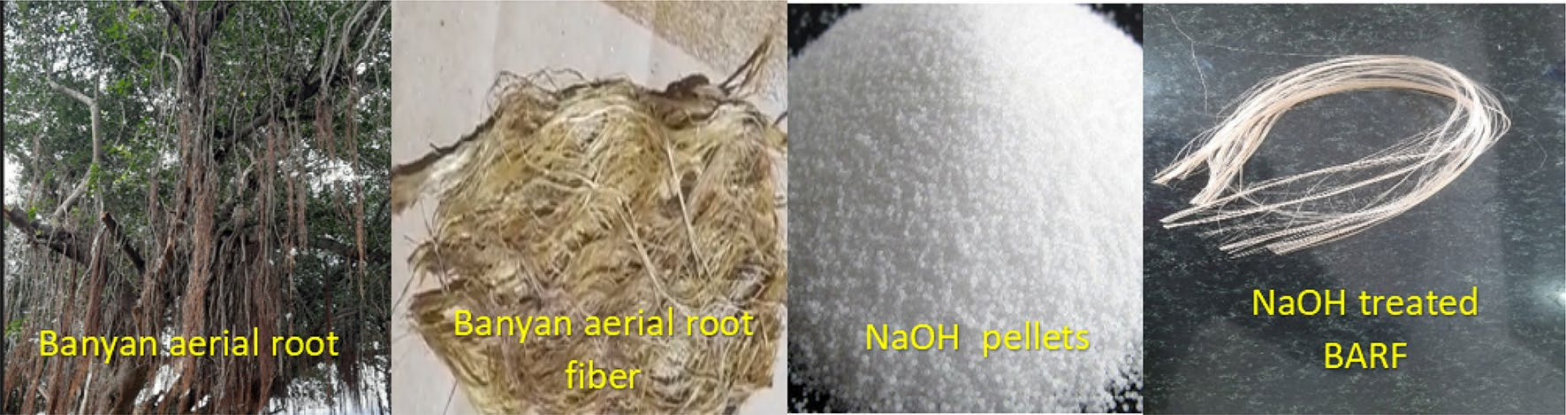
The extraction process of banyan aerial root fibre.

This study complies with relevant institutional, national, and international guidelines and legislation. Banyan aerial root fibre is a natural fibre sourced from a banyan tree in the village of Periyagaram in the Indian state of Tamil Nadu. The most common technique, water retting, involves submerging bundles of banyan aerial root stalks in water. When water gets to the stalk's core, it causes the inner cells to enlarge and burst through the outer layer, allowing more water and decay-causing bacteria to be absorbed. Sodium hydroxide was utilized for surface modification of alkaline treatment after the raw fibres were obtained by water retting and stitching^18^. A 10% sodium hydroxide solution was used to soak the extracted fibres, which had an average diameter of 0.241 mm, for samples A to D, followed by 30, 60, 90, 120 and 150 min at room temperature. To remove the alkali from the fibre's surface after the recommended soaking durations, a 2% acetic acid solution was used in a neutralization procedure^19^. After being neutralized, the fibre was washed with a 1% Sodium Bicarbonate solution and air-dried for 24 h to remove all moisture before testing^20^. Figure 1 shows the fibre extraction process from banyan aerial root and alkali-treated BAF.

### Testing of banyan aerial root fibre (BAF)

The fibre extracted from the banyan aerial root and the fibre surface was modified by alkaline treatment. After completion of fibre treatment, identifies the characterization of fibre, which will help to carry out the application part. The treated fibre was compared to an untreated control sample regarding its cellulose, hemicellulose, wax, lignin, moisture, and impurity levels. Therefore, the banyan fibre was conducted per ASTM standards for different characterization analyses: SEM, EDX, TGA, FTIR, XRD, and physical properties of fibre diameter and tensile strength. LEICA—Strellaris & CLSM microscope testing equipment is used for scanning electron microscopy examination to determine the microstructures surface of the treated banyan fibre and compare it to the structure of the untreated fibre. Morphological testing on metal-coated, completely dried, and alkali-cleaned banyan fibre (gold–palladium). The samples have been coated with metal and are now electrically conductible. This scanning electron microscope study employed an accelerating voltage of 5 kV. Energy Dispersive X-ray (EDX) microanalysis is an elemental (for instance, Carbon, Oxygen, and Nitrogen, etc.) analysis of natural fibres that are coupled with scanning electron microscopy and relies on the generation of characteristic X-rays that reveal the presence of elements present in the specimens^21^.

The mass loss of a single banyan fibre was then determined by utilizing ELTRA's TGA Thermostep apparatus to conduct a thermogravimetric investigation of the fibre's thermal stability throughout a 32 °C to 450 °C temperature range in the nitrogen inert gas mode. Sample preparation for the banyan fibre was done following the ASTM E1131 standard, and a 5 g particle was created. A platinum crucible was used to store the BAF while it was evaluated in the thermal analyzer. The test was conducted in a nitrogen gas atmosphere to exclude the possibility of oxidation of the BAF sample. All BAF samples had their kinetic activation energy determined. The treated fibre samples were analyzed using Fourier Transform Infrared Spectroscopy (FTIR) to determine the various functional groups. All measurements were taken using an experimental setup comprising a Perkin Elmer RXI spectrometer, scanning at 20 scans per minute with a resolution of 4 cm^−1^ in the wave number range 400 cm^−1^ to 5000 cm^−1^. Information on the existing functional groups was captured due to capturing the spectrum acquired from the experimental study^22^. The NaOH-treated banyan fibre (fibre form) was subjected to X-ray diffraction in Bruker-D8 Model to identify the crystalline structure and compounds present in the cellulose fibre as per the ASTM E43-49 standard used to experiment. The diffraction pattern and the X-ray diffraction method use a copper spectrum at a wavelength of 1.54 angstrom under reflection mode. The working voltage was set at 40 kV to establish the operating parameters. The equation used to calculate the crystallinity index from XRD results, CI (%) = (1– I _am_/I_200_) × 100, and crystalline size can be measured from the equation as CS = (k(λ/β).cos θ), the peak's Full width at half maximum (FWHM) is β, X-ray wavelength (1.54 nm), Scherrer's constant (k = 0.89), and diffraction angle^23^. The θ fibre diameter was determined using a DINO LITE digital microscope with a magnification of 220 ×. The tensile strength of treated banyan fibre was determined from UTM under a 3KW load tester as per the ASTM 638D standard and compared with an untreated tensile strength of banyan fibre^24^.

## Results and discussion

### XRD analysis of BAF

**Figure 2:**
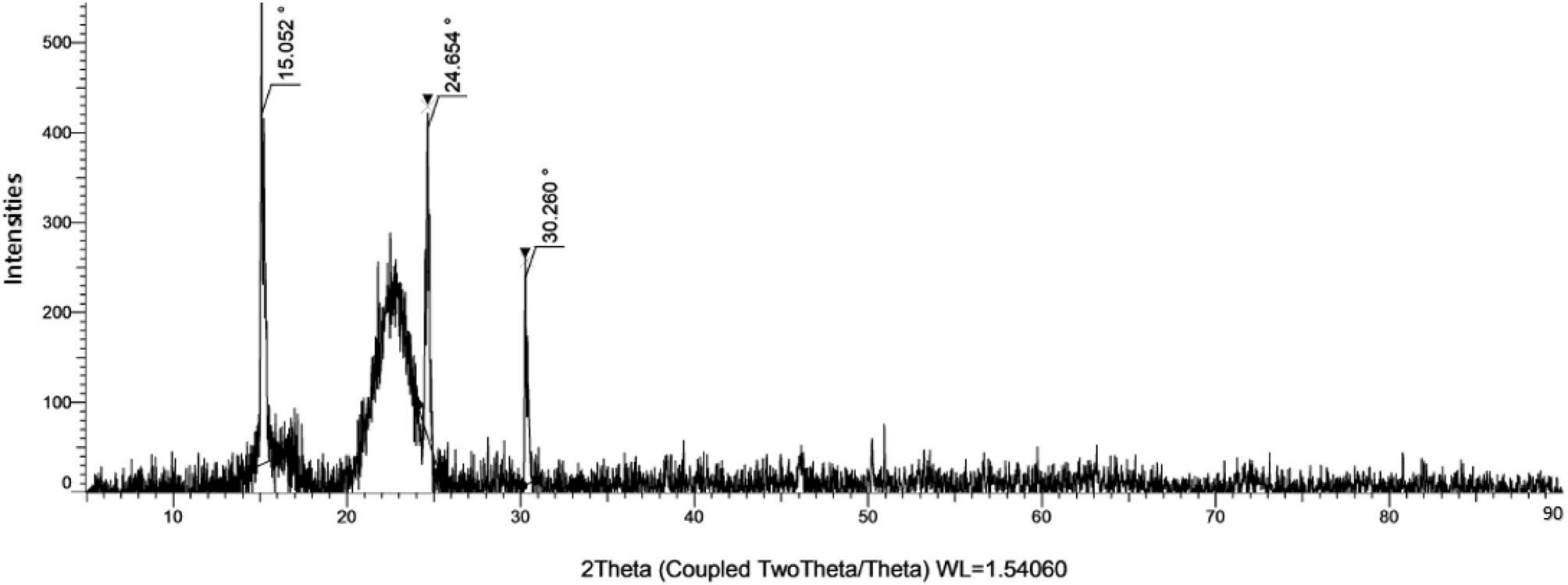
XRD graph of NaOH-treated banyan fibre.

Crystallite size and crystallinity index, two indicators of a fibre's crystalline nature, were measured and reported. X-ray diffraction analysis was performed^25^. Figure 2 displays the XRD graph for this banyan aerial root fibre. At the 2θ angles, the most prominent peaks are located at 15.052°, 24.654°, and 30.260°, with corresponding intensities of 447, 410, and 236, respectively. The broad cellulose peaks were observed from this XRD graph at 23° with an intensity of 217 counts, respectively.

The crystallinity index is 52% for this treated banyan aerial root fibre, 48% amorphous content was observed in this natural fibre, and the crystalline size of this treated banyan fibre is 51.2 nm. The CI for untreated banyan fibre identified from the literature is 39%, which shows clearly the chemical treatment is used to improve the characterization of the natural fibre of BAF. Another study used a conventional formula to determine the crystallinity index value, finding that it was 48.64%, much higher than the flax stem fibre FSF (42.92%) of untreated holy fig fibre but lower than those of treated banyan fibre^26^. Scherer's equation was used to determine that the crystallite size of treated BAF was 51.2 nm, an increase of 0.971 nm relative to the size of crystallites in untreated fibre. Increasing crystallite size can be attributed to the formation of soda celluloses, which can occur when an alkali solution penetrates the crystalline areas^7^. This opens the door for random breakage of the cellulose chains inside the crystalline domains.

### Tensile strength of BAF

**Figure 3:**
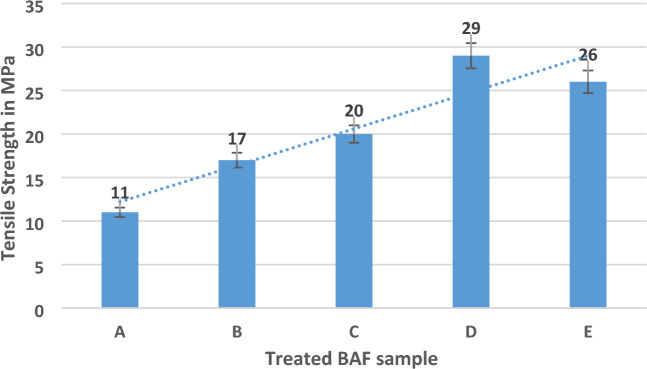
Tensile strength of BAF with different immersion periods.

All the structural application tensile test is mandatory due to the axial force requirement of the material. In this work, banyan aerial root fibre was extracted and treated with an alkaline solution at different periods as follows 30, 60, 90, 120, and 150 min. The results revealed a significant 120 min immersion time for this tensile strength of 29 MPa among all the other periods; 120 min was the maximum tensile strength for this BAF material. The primary reason behind these results is the natural fibre of banyan aerial root fibre can improve the fibre quality during this fibre treatment under sodium hydroxide solution for up to some period (120 min). Then it can reduce the strength due to the reduction of fibre strength under this NaOH solution, causing the removal of the impurities to 120 min duration. It starts to travel the internal molecules level of fibre when it reaches more time^26^. The results of tensile strength for this BAF as shown in Fig. 3. The fibre treatment at different periods was mentioned as samples A to E (30, 60, 90, 120, and 150 min), respectively.

### Thermogravimetric analysis of treated BAF

**Figure 4:**
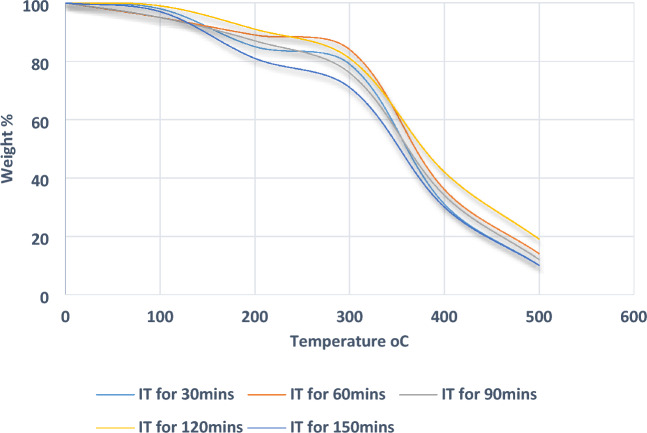
TGA curve for the different immersion periods of banyan fibre.

Figure 4 shows thermogravimetric analysis results of treated at different immersion periods of banyan fibre. The results revealed a mass at maximum temperature acquired in immersion time for 120 min and a minimum temperature of 30 min period. The initial peak is obtained for all the readings between 50 and 100 °C, and the second peak values are raised between 250 and 300 °C. The peak values are revealed in the first stage when low-temperature volatile materials are removed, and in the second stage, heat is absorbed, and the material starts to reach the fire point. Therefore, the TGA graphs revealed that this treated banyan fibre at 120 min immersion time could withstand more heat and reduce the mass loss for more temperatures. The untreated fibre TGA results from another research show that the peak values start at 40 to 80 °C, and the fire point reaching 260 °C can give clear evidence for the alkaline treatment used to improve the fibre under this thermal stability mode experiment. The treated FSF was shown to have much higher thermal stability than the untreated sisal fibre, as was seen in related studies. The treated sisal fibre immersed for 60 min exhibited the most incredible thermal stability compared to the other soaking intervals^27^.

The cellulose in the fibre starts to burn in the second stage due to the covalent bonding between the cellulose and fibre materials. Also, based on the TGA curve, the mass loss starts at 250 °C for the second peak is a significant fibre property that can work at high-temperature mode application for the structure materials. The thermal breakdown behaviour of cellulose fibres is drastically altered by alkali treatment. TGA analysis revealed that 24 h after being treated with 0.5% NaOH, the fibres had a moisture content of 6.17%, whereas untreated jute fibre had a moisture level of 11.32%. The hydrophobicity of jute fibre appears to have decreased as measured by TGA. This is because alkali treatment removes amorphous hemicellulose and lignin components from the fibres, making the treated fibres more thermally stable than the untreated fibres^28^.

### Physical analysis of BAF

**Figure 5:**
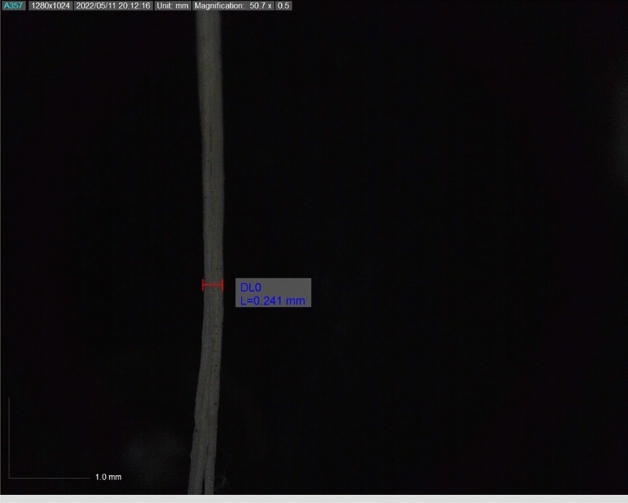
10% NaOH-treated banyan fibre microscopic image.

The physical analysis includes fibre diameter and density of treated fibre. The microscope measured the diameter of a single banyan fibre, as shown in Fig. 5. The untreated banyan fibre diameter is 0.29 mm which has been taken from the literature, and the treated fibre diameter was calculated for 120 min immersion time at a scale of 0.1 mm and the diameter is 0.241 mm 16% lesser than the raw banyan fibre diameter. The reason behind the reduction in diameter is, during the alkaline treatment, the removal of lignin, cellulose, and other impurities in the raw fibre^29^. The density of treated banyan fibre is 1.52 g/cm^3^, compared with untreated fibre, which is dense due to the reduced area and increased fibre mass due to this NaOH treatment. The increased density of FSF may be attributable to an increase in the comparatively dense -cellulose contents on the fibre surface.

### Fourier transform infrared (FTIR) analysis for BAF

The analysis result between wave number and normalized transmittance carries a frequency wave with significant peaks. There is an O–H group, which verifies the presence of cellulose. A C-H group verifies the presence of hemicellulose; a C = C group verifies the presence of wax; and a C–OH group verifies the presence of lignin, all based on the critical points^30^. The presence of cellulose components in the treated banyan fibre was confirmed by observing chemical bands associated with the hydroxyl chemical group at 2923 cm^−1^ and 1016 cm^−1^, just as they were in the raw BAF. Peaks verified the contribution of wax contents on the surface at 1583 and 1415 cm^−1^, which indicates the (CC bond) in the raw fibre. Since the carbonyl peak in untreated banyan fibre is located between 2362.74 and 2046.65 cm^−1^, it may be deduced that the hemicelluloses group was primarily removed from the fibre surface after the 10% NaOH treatment.

**Figure 6:**
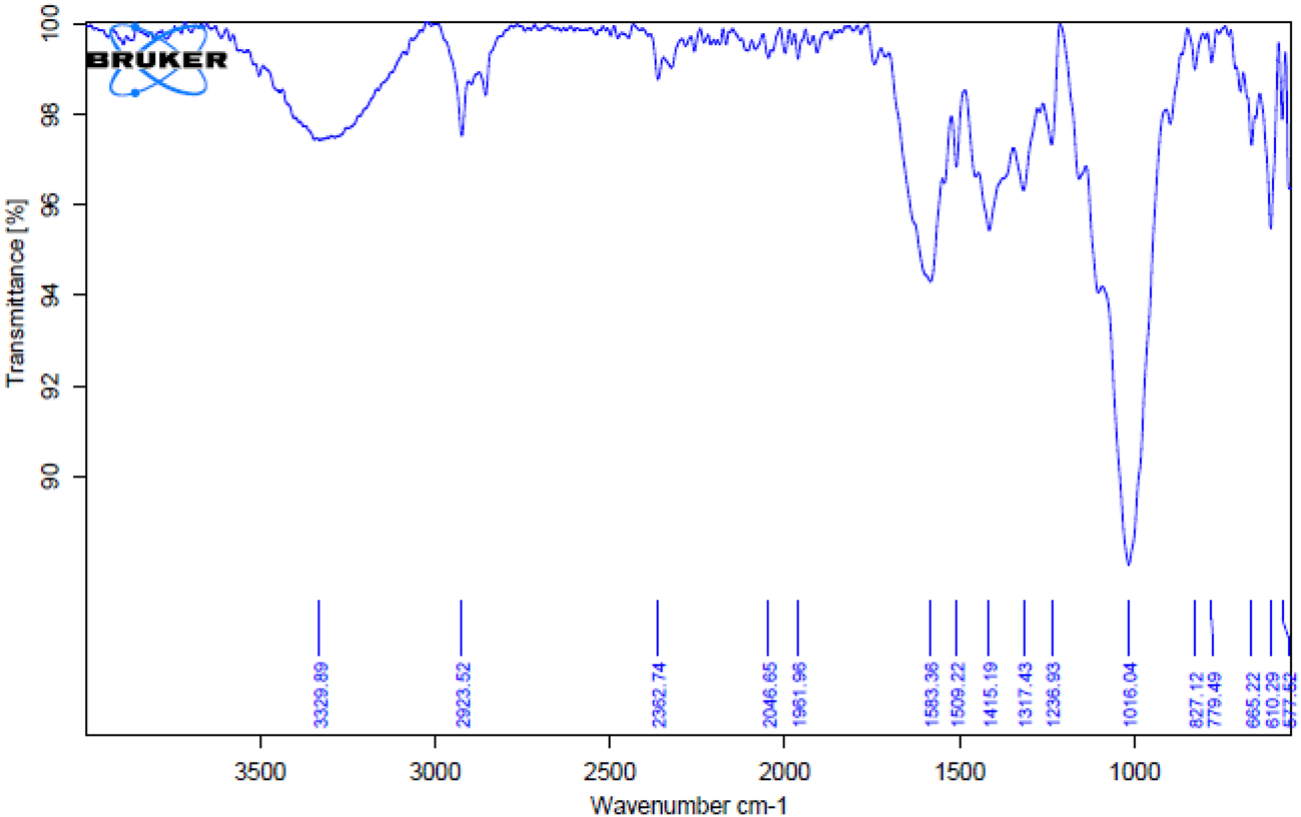
FTIR results of 10%NaOH treated banyan fibre.

Similarly, the 1740–1750 cm^−1^ peak in NaOH-treated hemp has been eliminated in another study. This is because the pectin and wax that naturally coated hemp fibres were stripped away. Untreated fibres peaked at 1100 cm^−1^ and 2850 cm^−1^, isolated following treatment. The interaction of NaOH with a small alcoholic group may illuminate the disappearance of the 1100 cm^−1^ peak, and the disappearance of the 2850 cm^−1^ peak during NaOH treatment was likely related to removing a methane group^30^. Alkali treatment's effect on hemicellulose removal cannot be seen in the FTIR plot. However, scanning electron microscopy photos will show that the treatment reduces the hemicellulose content on the fibre surface. The FTIR spectrum of banyan fibre treated with an alkaline solution is displayed in Fig. 6.

### SEM morphological analysis of BAF

**Figure 7:**
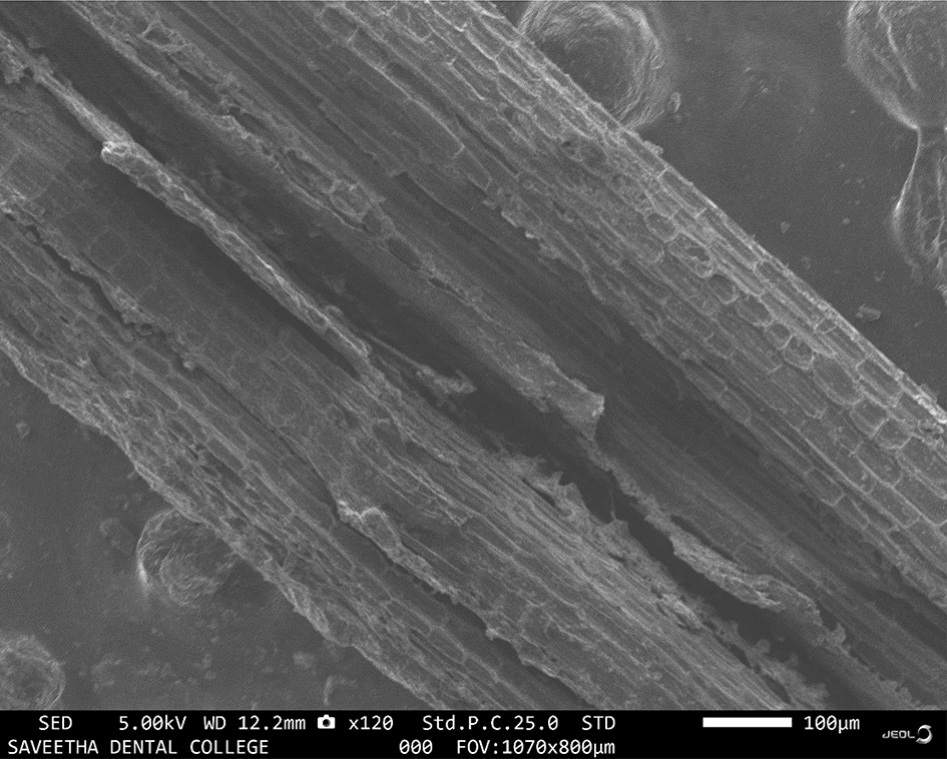
SEM image of NaOH-treated banyan fibre.

Fibre surface examination using a Scanning Electron Microscope (SEM) is conducted to learn more about the fibre. In addition to measuring the average fibre diameter with high magnification resolution, SEM allows us to readily visualize the distribution of elements present over the whole fibre surface. Researchers have shown that hemicellulose, lignin, and wax/impurity fibre content appear as white layers, a nail-like morphology, and tiny discontinuous sections, respectively^30^. The bonding characteristic of fibre can be identified as rough or smooth based on its appearance during bonding. Figure 7 shows an SEM image of NaOH-treated banyan fibre.

From the pictures, the fibre was spherical and packed with microfibres. It was hypothesized that waxes, grime, and oils were on the surface. Chemical processing of banyan aerial root fibres is required to manufacture the polymer matrix since this is where the contaminants and organic elements will be eliminated. The fibre's surface must be optimized for the polymer matrix's adherence.

### EDX analysis of BAF

**Figure 8:**
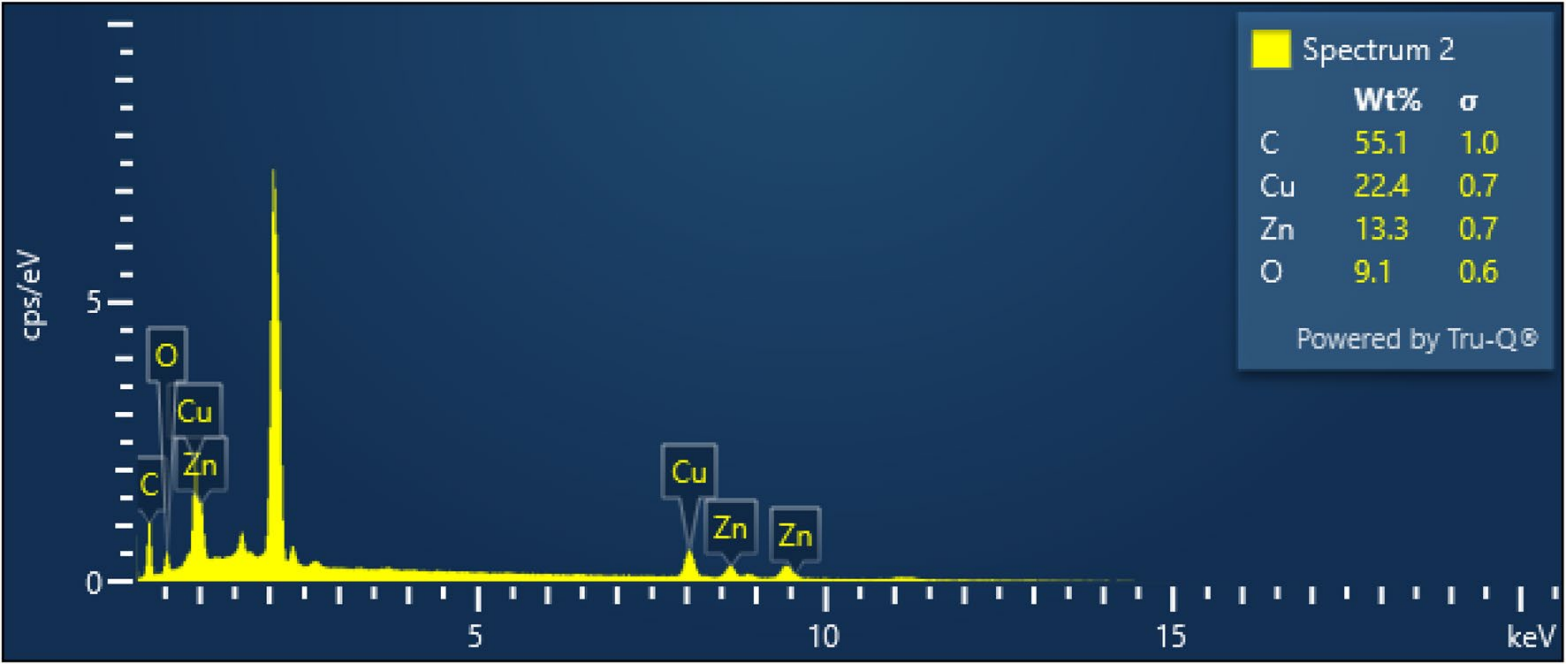
EDX graph of NaOH-treated banyan fibre.

This experiment used elemental analysis to determine the presence of carbon, oxygen, nitrogen, etc., in the treated banyan fibre. The weight and atomic percentage of the elements in the NaOH-treated banyan fibre were analyzed quantitatively. Carbon and oxygen make up the bulk of the fibre surface and are the fundamental components of BAF fibres. Carbon has a weight of 55.1% and an atomic percentage of 59.12, whereas oxygen has a weight of 9.6% and an atomic percentage of 36.97. Calcium, potassium, sodium, and magnesium, among the other elements, are depicted in EDX results. Another study found that palm fibre, another natural fibre, has 55.1% carbon and 22.4% copper, 13.3% zinc, and 9.1% oxygen, respectively. Hence the higher carbon and Cu content of banyan fibre demonstrates the former's superiority over the latter^31^. The EDX diagram of a banyan aerial root fibre is displayed in Fig. 8.

## Conclusion

Characterizing the fibre's surface is an essential first step in investigating its other properties, such as its physical, chemical, and tensile behaviour. Fibres can be extracted from leaves, stems, and roots, with the method chosen, depending on the researcher and the availability of extracting equipment. Using BAF treated with alkali proved suitable for structural purposes in the current study. The chemical analysis results indicated that the raw BAFs might have their 52% crystalline region and 48% amorphous components such as hemicellulose, lignin, wax, and other impurities significantly reduced by the 10 wt% NaOH alkali treatment with 120 min immersion time. The treatment of the BAFs with alkali was shown, by FTIR and XRD analyses, to be effective at removing the amorphous components. Maximum tensile was observed at 120 min immersion time. The result of tensile strength is 29 MPa. The TGA analysis showed that the surface modifications could improve the BAFs' 13% greater thermal stability. The surface nature of raw BAFs applied to NaOH chemical treatment was elucidated using SEM and EDX research. It is determined that the surface of natural fibres treated with 10wt.% alkali for 120 min may yield superior fibres that can be used to construct innovative composite materials for lightweight pollution-free applications such as car instrument panels.

### Future scope of this work

Natural fibre-based composites are better for the environment and can be used for various products, including cars, aerospace, ceiling panels, packaging, and more. Despite the fast destruction of forests worldwide, the demand for wood products continues to rise. Efforts are being made in green technology to develop wood substitutes that utilize wood materials blended with a polymer to create a low-priced, high-performance, and termite-resistant product.

## Data Availability

The datasets used and/or analyzed during the current study are available from the corresponding author on reasonable request.
